# Draft genome sequence of *Archaeoglobaceae* strain SR50 isolated from a shallow hydrothermal vent in the caldera of Saint-Paul Island, French Southern and Antarctic Lands

**DOI:** 10.1128/mra.00413-26

**Published:** 2026-07-30

**Authors:** Mélanie Le Moigne, Sébastien Le Guellec, Erwann Vince, Gaëtan Burgaud, Karine Alain

**Affiliations:** 1 Université de Brest, Ifremer, CNRS, EMR 6002 BIOMEX, BEEP, Plouzané, France; 2 Laboratoire Universitaire de Biodiversité et Écologie Microbienne, Université de Brest, INRAE, Plouzané, France; 3 Institut Universitaire de France 89211, Paris, France; University of Wisconsin-Madison, Madison, Wisconsin, USA

**Keywords:** *Archaeoglobaceae*, hydrothermal vent, lithoautotrophy, sulfate reduction, French Southern and Antarctic Lands

## Abstract

We report the draft genome sequence of SR50, the first cultivated member of the genus JdFR-22 within the *Archaeoglobaceae* family, which currently includes three genera with cultivated representatives. Isolated from a shallow hydrothermal vent off Saint-Paul Island, a strictly protected area, SR50 is able to grow lithoautotrophically by sulfate reduction.

## ANNOUNCEMENT

Saint-Paul Island, a geographically isolated and strictly protected area in the Indian Ocean (French Southern and Antarctic Lands), remains microbiologically poorly explored and likely represents a reservoir of taxonomic and genomic diversity.

Strain SR50 (UBOCC-M-3595) was isolated from a sediment-hydrothermal fluid sample collected at a 5 m depth vent in Saint-Paul Island’s caldera (SK23-SP-04; 38° 43′16.29″ S, 77° 31′21.69″ E; 23 December 2023). SR50 was isolated at 50°C through three successive one-tenth serial dilutions to extinction, in a mineral medium containing 20 mM Na_2_SO_4_, 25 g.L^−1^ NaCl, and an H_2_/CO_2_ (80%:20%) gas phase (1). The purity of the strain was confirmed by 16S rRNA gene and genome sequencing. Strain SR50 grows chemolithoautotrophically by sulfate reduction, producing sulfide while consuming sulfate, checked by colorimetric assays and ion chromatography (1).

Genomic DNA was extracted using the PCI method (1) and sequenced by Novogene using Illumina NovaSeq X Plus technology (2 × 150 bp paired-reads; Novogene-in-house kit; 13,429,174 raw reads). Raw reads were deposited on the Galaxy France server, and their quality was checked with FastQC v.0.12.1 (2). Adapter sequences were trimmed using Cutadapt v.5.2 (3), and reads were assembled using Unicycler v.0.5.1 (4). The ribosomal (rRNA) genes were extracted using barrnap v1.2.2 (5). Genomic annotation was performed using the MaGe platform v.3.18.2 (6). The 16S rRNA sequence similarity and average nucleotide identity (ANI) were calculated against sequences of the closest cultivated relatives using the EZBiocloud server (7). Average amino acid identity (AAI) scores (8) were determined using the AAI_calculator of Enveomics Gateway (https://enveomics.scigap.org) (9). Default parameters were used for all software.

The draft genome of strain SR50 consists of 85 contigs (2,366,160 bp, 39.44% G + C content), with a 63.62 × coverage and an N_50_ of 47,057 bp. Genome completeness was estimated at 99.99% with 0.15% redundancy using CheckM2 v.1.1.0 (10). It encodes 2,693 coding sequences, 45 tRNA, and 1 5S-16S-23S rRNA operon.

Using the GTDB-Tk classify workflow v.2.7.0 (11), strain SR50 was assigned to the *Archaeoglobaceae* family and the genus JdFR-22, which currently has no cultivated representatives. Its closest cultivated species are *Archaeoglobus sulfaticallidus* PM70-1^T^ (94.80% 16S rRNA similarity; OrthoANIu value of 67.56%; AAI score of 57.63%) and *Archaeoglobus veneficus* SNP6^T^ (94.73% 16S; 68.08% ANI; 62.43% AAI). Given that the 16S rRNA gene similarity values (94.55%–95.05%) fall within the range for new genus delineation, while ANI values (<95%–96%) and AAI values (<65%) are respectively below the species and genus thresholds (12, 13), these results indicate that strain SR50 likely represents a novel species and the first cultivated member of a new genus, referenced as *JdFR-22* in GTDB.

The genome encodes a complete dissimilatory sulfate reduction pathway (*sat*, *aprAB*, *dsrABCD*, *hmeABCDE*, and *qmoABC*), consistent with its ability to grow via sulfate reduction. Additionally, it encodes a sulfhydrogenase, a formate dehydrogenase, multiple multiheme cytochromes, a near-complete gluconeogenesis pathway, a complete C4-dicarboxylic acid cycle, and a near-complete reductive pentose phosphate cycle for carbon fixation. Only a few genes of the reductive acetyl-CoA pathway were identified. These genomic features highlight the similarities and differences between strain SR50 and other genera within the *Archaeoglobaceae* family (14–16).

## Data Availability

The Whole Genome Shotgun project has been deposited at GenBank/ DDBJ/ENA under the BioProject PRJNA1435636 and the accession number JBWLUP000000000. Fastq files of raw reads were deposited in National Center for Biotechnology Information Sequence Read Archive under accession number SRR37580810. Strain SR50 is available from the UBO Culture Collection (UBOCC; https://www.univ-brest.fr/ubocc) under number UBOCC-M-3595.
